# The Evolving Role of Information Technology in Haemovigilance Systems

**DOI:** 10.1155/2018/6183468

**Published:** 2018-03-08

**Authors:** Augusto Ramoa, Jorge Condeço, Maria Antónia Escoval, Jean-Claude Faber, Florentino Fdez-Riverola, Anália Lourenço

**Affiliations:** ^1^Instituto Português do Sangue e da Transplantação, IP, Rua de Bolama 133, 4200-139 Porto, Portugal; ^2^Department of Computer Science, ESEI, University of Vigo, Campus Universitario As Lagoas, s/n, 32004 Ourense, Spain; ^3^LuxConsulTrans, Banglamung, Thailand; ^4^Centro de Investigaciones Biomédicas (CINBIO), University of Vigo, Campus Universitario Lagoas-Marcosende, 36310 Vigo, Spain; ^5^Centre of Biological Engineering (CEB), University of Minho, Campus de Gualtar, 4710-057 Braga, Portugal

## Abstract

This work provides an overview and appraisal of the general evolution of IS/IT in haemovigilance, from which lessons can be learned for its future strategic management. An electronic survey was conducted among the members of the International Haemovigilance Network to compile information on the mechanisms implemented to gather, process, validate, and store these data, to monitor haemovigilance activity, and to produce analytical reports. Survey responses were analysed by means of descriptive statistics, and comments/observations were considered in the final discussion. The answers received from 23 haemovigilance organizations show a direct relationship between the number of collected notifications (i.e., communication of adverse effects and events) and the technical specifications of the haemovigilance system in use. Notably, IT is used in the notification reception of 17 of these systems, out of which 8 systems are exclusively based on Web solutions. Most assessments of the evolution of IS/IT tend to focus on the scalability and flexibility of data gathering and reporting, considering the ever-changing requirements of haemovigilance. Data validation is poorly implemented, and data reporting has not reached its full potential. Web-based solutions are seen as the most intuitive and flexible for a system-user interaction.

## 1. Introduction

The transfusion of blood and blood components is a critical procedure to consider when delivering healthcare services to patients. Haemovigilance aims to improve the safety of the blood supply by monitoring the entire value chain and contravening possible threats to the safety of transfusion recipients [1–3], with the recommendation of adequate corrective actions [4]. As such, haemovigilance information systems are required to give support to the monitoring of the safety of the blood supply to the organizations that provide or use blood products for patient treatment.

Among healthcare information systems, blood establishments and clinical pathology laboratories are considered pioneers in the use of information technologies (IT) [5]. Innovative efforts are mainly focused on improving the quality of the overall process responsible for the safe administration of blood components to patients [6]. Also, efforts have been done in increasing the efficiency of the blood chain process, namely, through a better management of the growing volume of information, a substantial reduction of paper records, the operation of mechanisms to decrease transcription errors, and a more efficient management of blood stocks [7]. For example, the implementation of labelling standards, such as the ISBT128, has enabled the unambiguous and language-independent identification of blood component units worldwide.

IT plays a key role in contributing to the safety and traceability of the transfusion chain, implementing/supporting the growing number of international guidelines and standards [8–13]. Current European Union legislation requires all member states to design and implement haemovigilance information systems to monitor the quality and safety of blood components for transfusion [13]. These information systems receive, process, and analyse notifications of transfusion reactions and adverse incidents occurring throughout the national blood transfusion chain, that is, from donor to recipient, where the healthcare professionals are responsible for such notification—hence the designation of notifiers.

These notifications consist in an extended set of questions that aim to characterize the adverse event or reaction that took place. For example, the notification of a patient adverse reaction should contain data about patient age, gender, diagnostics and reason for transfusing, symptoms and signs, blood component transfused, time and place of transfusion, reaction severity and imputability, type of transfusion reaction, and so forth.

Since the 90s, several national competent authorities (e.g., the Ministry of Health and the Health Authority) have created nationwide haemovigilance systems. In 1998, European countries founded the European Haemovigilance Network [14] with the purpose of exchanging best practices and benchmarks between the national haemovigilance systems [15]. Later, the International Haemovigilance Network (IHN) was created to accommodate non-EU members, becoming the single worldwide haemovigilance institution and a collaborating partner of the International Society of Blood Transfusion and the World Health Organization [16]. Nowadays, IHN has 29 nation members, namely, Australia, Austria, Belgium, Canada [17], Brazil [18], Croatia [19], Denmark, Finland, France [20], Germany, Greece, Iceland, Ireland [21], Italy [22], Japan, Luxembourg, Malta, Netherlands [23], Norway [24], New Zealand, Portugal, Singapore, Slovenia, South Africa, Spain, Sweden, Switzerland, United Kingdom [25], and United States of America [26].

Although different expert groups have publicly discussed both the events to be registered and the notification criteria these systems should comply with, little has been disclosed about their implementation and, most notably, the general evolution of IS/IT in haemovigilance. With the goal of gaining a better understanding about the IT involved in active and evolving haemovigilance systems, we conducted two anonymous Web surveys among the IHN members. These surveys collected information on the IS/IT portrait of the systems at two different points in time and focused on key aspects of the implementation of data registering, validation, and reporting. Also, they covered some primary aspects of data structuring and data security.

The rest of the paper is structured as follows: Section 2 describes the material and methods supporting these surveys, that is, the experimental design; Section 3 presents the results of the study, exposing several procedural and technological aspects; and Section 4 discusses the general evolution of haemovigilance systems and the main lessons learned. Finally, Section 5 provides some concluding remarks and outlines future work.

## 2. Materials and Methods

With the support of the IHN, two Web surveys were conducted among its members to collect information about the means used by their haemovigilance systems to collect data about patient and donor adverse reactions and events.

### 2.1. First Survey

The first survey aimed to obtain the first portrait of the haemovigilance system notification process. Therefore, this survey covered the main procedural aspects, notably the mechanisms of notification, the structure of the notifications, data management and analysis, and a general description of the IS/IT solution in use.

The mechanisms of reception of notifications were categorized into electronic (e.g., via e-mail or Web site), paper records (e.g., via fax or mail), or both simultaneously. In the case of Web-supported notifications, questions about the implementation included in-house versus outsourced development, the programming language, the database engine, and the inclusion of data safety measures. For the structure of the notification, attention was set on the use of plain, free-text descriptions, prestructured questionnaires with or without some free-text areas, and “guided” questionnaires, that is, where questions would be prompted according to previous answers.

Questions were made about the storage of notifications (e.g., the paper record was stored or the notification was transcribed), data validation (e.g., done automatically or by an expert), and reporting functionalities (e.g., transcribing data into spreadsheets or data access through the Web site).

The survey also covered the natural evolution of the systems, in particular, the dates of the system debut and its last update/revision, the number of staff members involved in haemovigilance management, and the number of registered institutions (healthcare facilities where blood is collected from donors and/or transfusion of blood components is performed), registered notifiers, and annual notifications received. Finally, the observation field allowed respondents to add any comments they considered relevant and not anticipated by the questionnaire.

A copy of this survey is presented in Supplementary 1.

### 2.2. Second Survey

Four years after the first survey, the same participants were asked to fill in a follow-up survey that accounts for any changes made to their systems, namely, in terms of the mechanisms of reception of notifications, the structure of the notifications, and notification management, validation, and reporting.

In both surveys, answers were received by an e-mail and through an Online Google Form, and results were anonymised.

A copy of this second survey is presented in Supplementary 2.

## 3. Results

**Figure 1:**
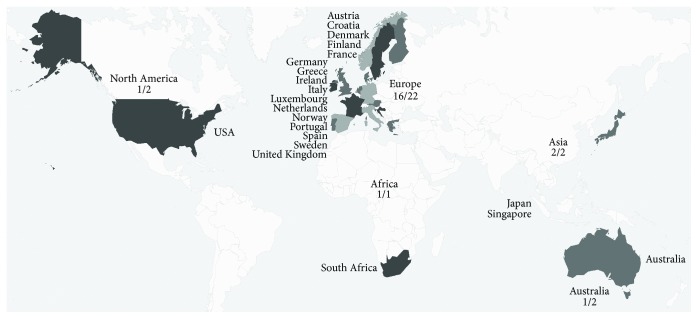
Distribution of IHN member responses.

The first survey was conducted between July 15 and November 7, 2013. At the end, we collected 23 answers from haemovigilance organizations of 21 different countries. While 21 countries might seem a small number at a global scale, they represent a response rate from IHN members of 72.4% (Figure 1). The fact that haemovigilance is a relatively new area and that IHN is the worldwide reference in this field grants that this response number is quite significant.

Two of the IHN members that participated in the survey indicated that they maintain separate data management systems for patient and donor notifications, and another participant indicated that the corresponding data management system does only record the notifications of a region of the country.

Concerning the institutions and notifiers registered in the system, as well as the number of annual notifications, the participants described very different operational realities. For comparison purposes, the extent of the system is considered directly related to the number of annual notifications. Systems were grouped in the following intervals: less than 50, between 51 and 100, between 101 and 250, between 251 and 500, and more than 500 notifications per year.

**Figure 2:**
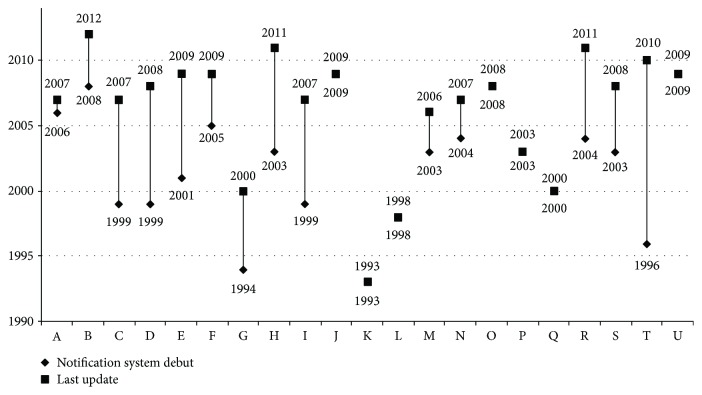
Chronology of the debut and last update of the haemovigilance systems that responded to the first survey. Letters A to V represent each system.

Regarding longevity, 31.8% of the haemovigilance systems were implemented between 1993 and 1999 (Figure 2). The year when a larger number of debuts was registered was 2003 (18.2%), and the last system launch occurred in 2009. Moreover, participants declared that 63.6% (14 of the 22 systems) of these haemovigilance systems have been redesigned from an infrastructure reengineering perspective. The oldest system, which started its activity in 1993, still maintains the original notification method, but the respondent stated in the observation field that a new electronic version is going to be released shortly.

The number of people enrolled in the haemovigilance system, namely, IT staff, office workers, and scientific staff, grows as the number of notifications increases. In systems receiving 50 or less notifications per year, the average number of staff is 1.0. For systems dealing with hundreds or more notifications, the average number of staff visibly increases: 1.8 in systems receiving between 101 and 250 notifications, 4.7 in systems receiving between 251 and 500 notifications, and 7.0 in the larger systems.

Looking into the IT staff of the larger systems, the 5 larger systems that do not use a Web site have a low average of IT staff (0.8); in the 6 larger systems that have a contracted Web site, this number almost doubles (1.58); and in the 2 larger systems, which developed their own Web site, the average of IT staff is 0.75.

**Figure 3:**
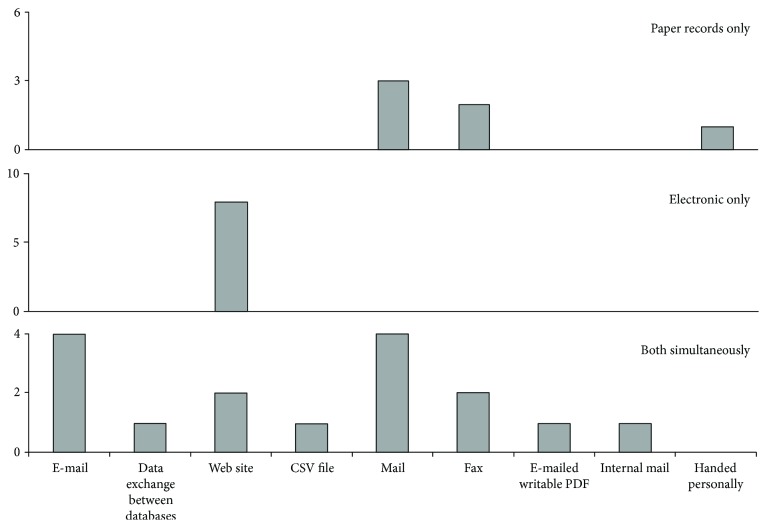
Categorization of haemovigilance systems according to the ways they collect notifications: systems that receive paper record notification, those that receive electronic notification, and those that receive electronic and paper record notifications simultaneously. There is one more haemovigilance system that uses a Web site, but we excluded it from this chart, since it has not answered the question regarding what type of notification reception methods was used.

Focusing on the notification method, 23.8% of the 21 responses receive only paper record notifications (one of the systems receives notifications both by a mail and by being handed personally), 38.1% receive only electronic notifications, and 38.1% receive both (paper record and electronic notifications) simultaneously.  Figure 3 details the reception methods used. Noteworthy, the systems that rely only on electronic notifications use the Web site as means of submission.

Tables 1 and 2 summarize the collected data regarding notification reception methods and the structure of the notifications.

In 45.5% of the systems, the notifications are automatically stored; that is, the notification record is directly stored in the database. Regarding the rest, 4.5% of the systems transcribe (e.g., to an Excel spreadsheet) the notifications, 18.2% of the systems store the notification in paper records, 22.7% of the systems store and transcribe the notifications, and the remaining 9.1% of the systems have a mixed system (some notifications are automatically stored in a database and others are transcribed). Automatic data validation is fully implemented in only 9.1% of the systems and is partially implemented in 13.6% of the systems. In turn, 68.2% of the systems validate the notification manually, and apparently, the remaining 9.1% do not validate the notifications.

Reporting and analysis tools are fully integrated in 27.3% of the haemovigilance systems, data importation is supported in 31.8% of the systems (e.g., database connection or file upload), and manual transcription of data to third-party analytical software like SPSS or Excel is used in most systems (40.9%).

Finally, the predominant database solutions are Oracle (36.4%) and MySQL (36.4%) database management systems. 18.2% of the systems use Microsoft SQL Server or InterSystems Caché, and 9.1% of the participants did not specify the database engine in use. The most common Web programing languages are JavaScript (31.8%), PHP/HTML (27.3%), Java (13.6%), and C, C++, or C# (9.1%). The remaining systems indicated the use of .NET, InterSystems Caché Object Script, XML, and webMethods or did not know such specifics.  Table 3 summarizes the protection mechanisms implemented by the systems. The maximum number of measures is 7 (one system), and the minimum number of measures is zero (one system). Login-based access, password protection, data privacy, and data anonymity are the measures more frequently implemented.

The second survey took place between September 18 and 27, 2017, and aimed to identify recent or soon-to-happen developments and/or procedural changes. Half of the respondents stated no changes in the haemovigilance systems in the 4-year interval between surveys. Three haemovigilance systems have changed the notification procedures: the first has replaced a word document, the second transcribes the received data to a database through a specific software, and the third now collects data directly from their notifiers. One haemovigilance system has changed the notification structure, replacing free-text questions by check boxes (i.e., avoiding free-text descriptions), and the other is implementing “adaptable questions,” that is, questions placed according to previous answers.

Among the systems maintaining paper records, one has started digitizing these records and storing the obtained PDF in a database. Also noticeably, one system has incorporated benchmark software to give feedback to the notifiers, namely, to obtain summary reports.

In terms of the validation of notifications, the most significant developments are the manual validation on all notifications now implemented in one of the systems and the testing of an “external validation method” in another system.

## 4. Discussion

This survey offers a unique view of the IS/IT landscape, and general practices, of the haemovigilance systems worldwide. The high rate of response (72.4%) reflects the strong involvement and interest of IHN members in these matters.

The surveyed systems present a great heterogeneity, most notably in terms of the number of notifications received and the number of notifiers and registered institutions. Arguably, this variability can be explained by the intricacies of the transfusion chain activity and by the level of maturation of the systems at the national level. For example, we notice the existence of countries with less than 10 million habitants where their haemovigilance system receives more than 500 notifications per year, while others with more than twice the population receive half of these notifications. These statistics could be the subject of further study, trying to correlate the number of notifications with the transfusion chain activity, the gross domestic product spent on health (a possible outcome of such a study could confirm or refute that less investment in healthcare may lead to less attention/effort put on these matters), the rate of population, and the number and type of professionals working in the transfusion area, among others.

### 4.1. Notification Data Reception and Management

Generally, haemovigilance systems that receive a larger number of notifications have computer-assisted solutions. When the system receives only electronic notifications (40%), the Web is the preferred means of user-system interaction. Actually, 25% of the 12 systems that still use paper records stated that the implementation of electronic notification procedures will be conducted in the near future.

Regarding the structure of the notification, only 1 participant uses a “tailored” method where questions are placed according to previous answers, while all the others pose a predetermined set of questions, which may not always apply to the notified event.

Probably, the reduced number of systems performing the automatic validation of the notifications (only 9%) reflects the lack of control over the submitted data. A paper notification that is faxed to the haemovigilance system must be examined to check if all required questions were answered, whereas in a Web-based system, the form submission can be blocked if the required questions are not answered. Moreover, the Web form analyses the answers as they are inserted, guiding the notifier through the notification. As an example, a Web-based system can hamper a notifier from classifying an adverse reaction as “febrile nonhaemolytic reaction” if the symptom “fever” was not previously selected. In a system with automatic validation, the number of notifications that require expert supervision will certainly be lower.

Automatic analytical reporting is also poorly implemented. Only 26% of the systems automatically generate reports, whereas the rest rely on human resources to import or transcribe the data to third-party software for further processing.

### 4.2. IT Details

IT investment is economically affordable. Most of the existing systems use technologies that are free of charge, namely, MySQL, PHP, HTML, JAVA, and JavaScript. Furthermore, the size of the IT team seems to be related to the volume of data that the haemovigilance system is required to manage, notably the number of notifications, and unrelated to the method of collecting notifications; that is, even in systems with no Web interface, IT staff is still necessary for subsequent management/analytical tasks like data importing, handling, and reporting.

Overall, electronic data storage, computer-aided data validation, and automatic generation of analytical reports represent the added value of nowadays IT-based haemovigilance systems, both at operational and management levels. These modules support daily routines, reducing the time and human effort involved, whereas enforcing data quality and enabling decision-making. The existence of open-answer questions is an example, as they require more attention by haemovigilance experts.

On another point, we would like to raise awareness to in-house development and the enforcement of general safety measures. Survey results show that in-house-developed systems (F, G, H, and I in Table 3) have an average of 2.5 general safety measures, while outsourced systems (A, B, C, D, E, J, and K in Table 3) have an average of 5 general safety measures. This is a matter that, in our opinion, deserves improvement.

### 4.3. Functionalities Implemented Since 2013

The data collected on the second survey shows that 50% of the systems did not experience significant developments in 4 years. The changes implemented in the other systems aim for the reduction of paper records, modifications in the notification forms (i.e., replacement of free-text questions by multiple-choice questions), and the transcription of information to other software for further reporting.

## 5. Conclusions

After collecting insights into the experience and opinion of those closely connected to the management of national haemovigilance systems worldwide, it is clear that a significant number of haemovigilance systems have invested in the elimination of paper records and the reduction of free-text questions in notification forms, that is, the automatic reception of notifications and the enforcement of the quality of these notifications. There are an increasing number of systems choosing Web-based solutions, and there is an increasing interest in developing analytical functionalities, namely, to comply with national and European legislation.

Although some of the haemovigilance systems still use nonelectronic notification systems, current developments and feedback from national administrators pinpoint system interconnection and data interoperation/sharing as the future milestones for IS/IT solutions in haemovigilance. The rationale is that sharing data across systems would result in higher data completeness and consistency, enabling the generation of standardized, real-time reports about relevant quality and safety indicators for the European Commission and similar organizations. Therefore, national haemovigilance systems could benefit from stronger international guidelines for the implementation and maintenance of their notification process.

### 5.1. Future Directions

It is our belief that the discussion of large-scale, integrative data management approaches, namely, data federation and data warehousing systems, would push towards the development of European or international data repositories. An analysis of the data collected until now could lead to the replacement of open-answer fields by multiple-choice questions. For example, instead of asking to describe the signs and symptoms, put a list of them, basing this list in the ones described until now.

## Figures and Tables

**Table 1:** Notification reception methods. While paper records (either exclusively or simultaneously with electronic notifications) are present in systems from all sizes, all exclusively electronic interfaces are found in systems with more than 250 annual notifications.

Reception method	Number of annual notifications				
	≤50	51–100	101–250	251–500	>500
Paper records only	1			1	3
Both simultaneously	1		2	1	4
Electronic only				2	6

**Table 2:** Structure of the notification. 90.9% of the participants receive notifications using prestructured questionnaires.

Structure of the notification	Number of annual notifications				
	≤50	51–100	101–250	251–500	>500
Prestructured questionnaire	1				2
Prestructured questionnaire and free-text areas	1		2	4	9
Questions placed according to previous answers					1
No answer					1

**Table 3:** Safety measures implemented by the surveyed haemovigilance Web sites.

Safety measure	Web-based information system											Total
	*A*	*B*	*C*	*D*	*E*	F	G	H	I	*J*	*K*	
Access to site is password protected	*Y*	*Y*	*Y*	*Y*	*Y*	N	Y	Y	Y	*Y*	*Y*	10
Data privacy, that is, each participant is granted access only to his/her data	*Y*	*N*	*Y*	*Y*	*Y*	N	Y	Y	N	*Y*	*Y*	8
Data anonymity, namely, by masking the identification of clinical facilities, patients, and so forth	*Y*	*N*	*Y*	*Y*	*Y*	N	Y	N	Y	*Y*	*Y*	8
Data operation conditioned by user credential	*Y*	*N*	*Y*	*Y*	*N*	N	Y	Y	N	*Y*	*Y*	7
User sessions	*Y*	*N*	*Y*	*Y*	*N*	N	Y	N	N	*Y*	*Y*	6
Secure HTTP	*Y*	*N*	*N*	*Y*	*Y*	N	N	N	N	*N*	*Y*	4
Encryption of sensitive data	*N*	*N*	*N*	*Y*	*N*	N	N	N	N	*N*	*N*	1
Authentication with a national professional health card	*Y*	*N*	*N*	*N*	*N*	N	N	N	N	*N*	*N*	1
Total of safety measures implemented	*7*	*1*	*5*	*7*	*4*	0	5	3	2	*5*	*6*	

Letters A to K represent the haemovigilance systems. The columns referring to outsourced systems are in italic.

## Supplementary Materials

Supplementary 1 A copy of the first survey.

Supplementary 2 A copy of the second survey.
