# Supplementary material for: The Evolving Role of Information Technology in Haemovigilance Systems
Source: J Healthc Eng. 2018 Mar 8;2018:6183468. doi: 10.1155/2018/6183468 (PMC5863304; doi:10.1155/2018/6183468)
Supplement: Supplementary 1 — A copy of the first survey. [file 6183468.f1.pdf]

Haemovigilance Survey

Please note that all responses should reflect the current situation.

*Required

1. **Country** *

____

2. **Person responding** *

____

3. **Institution**

____

4. **Position**

____

Haemovigilance notifications

5. **How do you receive the notifications?** *
Mark only one oval.

- ( ) Electronically *Skip to question 6.*
- ( ) In paper records *Skip to question 7.*
- ( ) Both *Skip to question 8.*

Electronic notifications

6. **How do you receive the electronic notifications?** *
Mark only one oval.

- ( ) E-mail *Skip to question 14.*
- ( ) Website *Skip to question 10.*
- ( ) E-mail and Website *Skip to question 10.*
- ( ) Other: _______________________ *Skip to question 14.*

Skip to question 10.

Paper record notifications

7. **How do you receive the notifications?** *
Tick all that apply.

- [ ] Fax
- [ ] Mail
- [ ] Handed personally
- [ ] Other: ____________________

Skip to question 14.

Notifications

8. **Electronic notifications** *
Mark only one oval.

- ( ) E-mail *After the last question in this section, skip to question 14.*
- ( ) Website *After the last question in this section, skip to question 10.*
- ( ) E-mail and Website *After the last question in this section, skip to question 10.*
- ( ) Other: ____________________ *After the last question in this section, skip to question 14.*

9. **Paper record notifications** *
Tick all that apply.

- [ ] Fax
- [ ] Mail
- [ ] Handed personally
- [ ] Other: ____________________

Website

10. **What programming languages are in use** *
If possible, confirm with the web platform administrator
Tick all that apply.

- [ ] HTML / PHP
- [ ] JavaScript
- [ ] Java
- [ ] ASP
- [ ] .NET
- [ ] ColdFusion
- [ ] WebDNA
- [ ] Flash
- [ ] C, C++ or C#
- [ ] Ruby
- [ ] Unknown
- [ ] Other: ____________________

11. **What database is in use?**

If possible, confirm with the database administrator

Mark only one oval.

- ( ) MySQL
- ( ) Microsoft SQL Server
- ( ) Oracle
- ( ) Unknown
- ( ) Other: ____________________

12. **Is your software developed in-house?**

If not, please choose other and identify the company responsible for the software

Mark only one oval.

- ( ) In-house
- ( ) Other: ____________________

13. **Regarding safety measures, which one(s) are implemented in your software?**

If possible, confirm with the system administrator

Tick all that apply.

- [ ] Access to site (or parts of it) is password protected
- [ ] User sessions
- [ ] Data operation is conditioned by user credential
- [ ] Data privacy, i.e. each participant is granted access only to his data
- [ ] Data anonimity, namely by masking the identification of clinical facilities, patients, etc
- [ ] Secure HTTP
- [ ] Other: ____________________

Type of notification

14. **How is the notification formulated?** *

Mark only one oval.

- ( ) Plain text describing the situation
- ( ) Pre-structured questionnaire
- ( ) Pre-structured questionnaire and free text areas
- ( ) Questions placed according to previous answers

Notification management

15. **What do you do with the received notifications?** *

Tick all that apply.

- [ ] I transcribe the notification (e.g. to a Excel spreadsheet)
- [ ] I store the received notification (e.g. store original paper record)
- [ ] The notifications are automatically stored (e.g in a web database)

16. **How do you validate the notifications?** *

Mark only one oval.

- ( ) I don't validate the notification
- ( ) I must check the notification fully
- ( ) I partially check the notification
- ( ) Notifications are validated automatically

Reporting

17. **How do you obtain a summary report?** *

Tick all that apply.

- [ ] I transcribe data manually to a computer software (Excel, SPSS, Access,...)
- [ ] I import data, loading files or accessing a database, into a computer software (Excel, SPSS, Access,...)
- [ ] I obtain it automatically

Characterization

18. **Since when is your haemovigilance system implemented?**

____

19. **Since when is your current notification reception/handling method implemented?**

____

20. **How many institutions are registered in your database?**

Number of blood establishments, hospital blood banks and similar institutions registered

Mark only one oval.

- ( ) 50 or less
- ( ) Between 51 and 100
- ( ) Between 101 and 250
- ( ) Between 251 and 500
- ( ) More than 500
- ( ) Institutions are not registered

21. **How many notifiers are registered in your database?**

Number of persons authorized to notify

Mark only one oval.

- ( ) 50 or less
- ( ) Between 51 and 100
- ( ) Between 101 and 250
- ( ) Between 251 and 500
- ( ) More than 500
- ( ) Notifiers are not registered

22. **How many notifications do you receive annually?**

Number of notifications (donor and patient) received

Mark only one oval.

- 50 or less
- Between 51 and 100
- Between 101 and 250
- Between 251 and 500
- More than 500

How many persons are involved in the haemovigilance management?

Number of persons of your national authority involved in haemovigilance

23. **Office worker**

clerk, receptionist, typist, ...

24. **Information Technology staff**

Systems engineer, IT technician,...

25. **Scientific staff**

Persons involved in validating notifications, training and supporting notifiers

26. **Observations/notes/suggestions you wish to include**

Please mention also if any future change to the way you receive and handle notifications is being considered

Powered by
Google Forms
