# Supplementary material for: The Evolving Role of Information Technology in Haemovigilance Systems
Source: J Healthc Eng. 2018 Mar 8;2018:6183468. doi: 10.1155/2018/6183468 (PMC5863304; doi:10.1155/2018/6183468)
Supplement: Supplementary 2 — A copy of the second survey. [file 6183468.f2.pdf]

Haemovigilance Survey #2

In 2013 the Portuguese Haemovigilance System conducted a Survey on the Use of Information Technologies by the Haemovigilance Systems and we would like to start by acknowledging your participation.

After 4 years, we are resuming this study and one more time we would like to ask your precious collaboration one more time on this 2-page survey.

*Required

1. **Country** *

____

2. **Person responding** *

____

3. **Institution**

____

4. **Position**

____

Adjustments since 2013

We would like to know if your system suffer any significant changes since the previous Survey (Late 2013).

If yes please state them in the "Other" field

5. **Are there any changes in the way you receive the haemovigilance notifications (e.g. paper record notifications replaced by PDF sent by e-mail, new web form,...)?** *

   Mark only one oval.

   - ( ) No
   - ( ) Other: _______________________

6. **Are there any changes in the type of notification (e.g. implemented a Pre-structured questionnaire, added/removed free-text areas, adaptable questionnaire where questions placed according to previous answers,...)?** *

   Mark only one oval.

   - ( ) No
   - ( ) Other: _______________________

7. **Are there any changes in the way you process the received notifications? (e.g. we no longer keep paper records, we transcribe the notifiction to a software, the notifications are stored in a database,...)?** *

   Mark only one oval.

   - ( ) No
   - ( ) Other: _______________________

8. **Are there any changes in the way you validate notifications? (e.g. Notification validation is made automatically/manually/not done,...)?** *

Mark only one oval.

- No
- Other: ____________________

9. **Are there any changes in the way you obtain a summary reports? (e.g. Data is gathered into a computer software by loading files or accessing a database, summary report is obtained automatically...)?** *

Mark only one oval.

- No
- Other: ____________________

10. **Are there any other comments you wish to share with us?**

____

____

____

____

____

Powered by
Google Forms
